# Ab Initio Screening of Doped Mg(AlH_4_)_2_ Systems for Conversion-Type Lithium Storage

**DOI:** 10.3390/ma12162599

**Published:** 2019-08-15

**Authors:** Zhao Qian, Hongni Zhang, Guanzhong Jiang, Yanwen Bai, Yingying Ren, Wenzheng Du, Rajeev Ahuja

**Affiliations:** 1 Key Laboratory for Liquid-Solid Structural Evolution and Processing of Materials (Ministry of Education), Shandong University & Shenzhen Institute of Shandong University, Shenzhen 518057, China; 2 Shandong Management University, Jinan 250100, China; 3 Condensed Matter Theory Group, Department of Physics and Astronomy, Ångström Laboratory, Uppsala University, SE-75237 Uppsala, Sweden

**Keywords:** conversion electrode, doping design, lithium storage, light metal hydrides, density functional theory, electronic structures

## Abstract

In this work, we have explored the potential applications of pure and various doped Mg(AlH_4_)_2_ as Li-ion battery conversion electrode materials using density functional theory (DFT) calculations. Through the comparisons of the electrochemical specific capacity, the volume change, the average voltage, and the electronic bandgap, the Li-doped material is found to have a smaller bandgap and lower average voltage than the pure system. The theoretical specific capacity of the Li-doped material is 2547.64 mAhg^−1^ with a volume change of 3.76% involving the electrode conversion reaction. The underlying reason for property improvement has been analyzed by calculating the electronic structures. The strong hybridization between Lis-state with H s-state influences the performance of the doped material. This theoretical research is proposed to help the design and modification of better light-metal hydride materials for Li-ion battery conversion electrode applications.

## 1. Introduction

With the challenge of environmental problems and the consumption of fossil fuels, the demand for environmentally friendly energies has stimulated more and more scientists and engineers to develop renewable and green energies all over the world. In recent years, some renewable energy sources such as solar energy and wind energy have been developed rapidly [1,2,3]. The electricity generated by these clean energy sources can be either on grid or stored in chemical energy temporarily. For the chemical energy storage, the Li-ion battery has been regarded as one of the candidate technologies and has been a hot topic.

In the past years, a great number of researchers had been devoted to improving the properties of Li-ion batteries and achieved great progress, while Li-storage based on the traditional intercalation/deintercalation mechanism has limited the further development of the technology. It is still facing challenges to meet the increasingly high industry demands. A decade ago, Oumellal et al. put forward a new kind of Li-ion battery which uses metal hydride as the negative electrode [4]. This idea is based on the conversion reaction MH*_x_* + *x*Li^+^ + *x*e^−^ = *x*LiH^+^ + M and has a higher capacity density than general intercalation-type electrodes. The concept has paved a new way for the development of Li-ion battery materials [5,6,7,8,9].

Mg(AlH_4_)_2_ is a kind of lightweight hydrogen storage material with a hydrogen content of 9.3 wt.%. It has a fair capacity density while its thermal dehydrogenation/rehydrogenation is slow [10,11,12,13,14,15]. Traditional studies focused on how to improve the kinetics of the dehydrogenation/rehydrogenation reactions [16,17,18,19,20,21,22,23] and few researchers combined the complex hydride with the metal-ion battery. A recent publication reported the application of Mg aluminate in Mg batteries [24]. It is believed that the electrochemical usage of this complex metal hydride with more than one kind of chemical bonding (compared with the simple binary hydride) could be a new perspective for energy storage. While, in this work, we consider the atomic and electronic structures of pure and various doped Mg(AlH_4_)_2_ systems to explore and screen their potential applications in Li-ion battery for the first time and make attempts to improve the complex hydride’s performance through quantum mechanical first-principles design. This theoretical study aims at deepening the understanding of Mg(AlH_4_)_2_ system, advancing its new utilization as conversion electrode material for lithium storage and creating guidance for the experimental development of the material.

## 2. Methods

The structural relaxations and total energy calculations were performed using the projector augmented wave (PAW) method [25] based on the density functional theory (DFT) [26] by using Vienna ab initio simulation package code. The scheme of Perdew–Burke–Ernzerhof (PBE) was employed for the exchange-correlation functional in the generalized gradient approximation (GGA) [27] and a plane wave basis set with an energy cutoff of 520 eV was used to describe the electronic wave function. The K-point mesh of 7 × 7 × 5 was tested and employed for the Mg(AlH_4_)_2_ 2 × 2 × 2 supercell. The geometry optimizations have been done by minimizing the forces on atoms until the force on each ion is lower than 0.01 eV/Å with a conjugate gradient (CG) algorithm and, meanwhile, minimizing stress on unit cells without any symmetry constraint.

## 3. Results and Discussion

**Figure 1:**
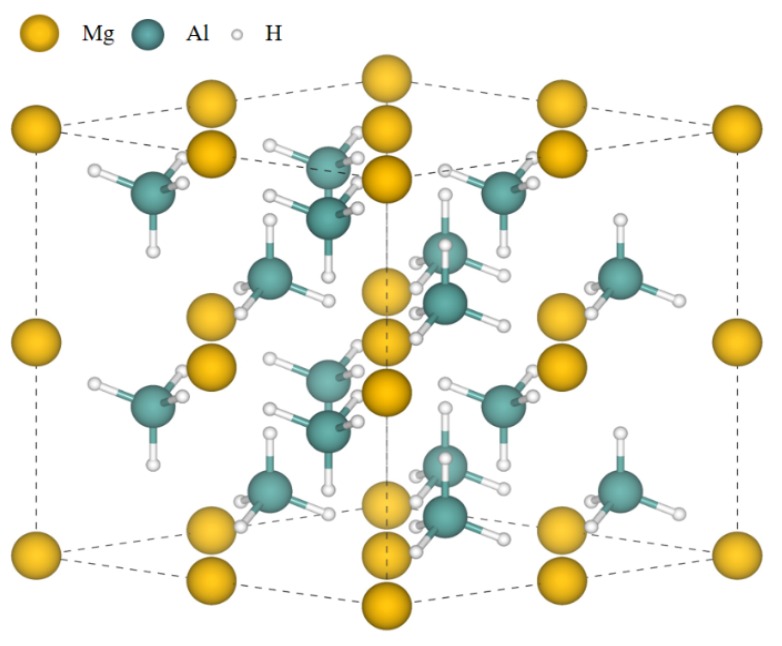
The atomic structure of Mg(AlH_4_)_2_ after structural relaxation. Mg atoms are in yellow; Al atoms are in blue; H atoms are in white.

The Mg(AlH_4_)_2_ phase is in a hexagonal structure that belongs to P3m1 space group. To calculate the atomic structure accurately, the unit cell has been expanded into a 2 × 2 × 2 supercell with geometrically optimized lattice parameters: *a* = *b* = 10.35 Å, *c* = 11.75 Å; α = β = 90°, and γ = 120°. The space group was confirmed to be P3m1 with single unit cell dimensions of *a* = 5.2084(3) Å and *c* = 5.8392(5) Å at 8 K, *a* = 5.20309(12) Å and *c* = 5.8400(2) Å at 111 K and *a* = 5.1949(2) Å and *c* = 5.8537(2) Å at 295 K in the experimental values [28]. In each supercell shown in Figure 1, there are 8 Mg atoms, 16 Al atoms, and 64 H atoms. For this structure, all the atoms are divided into one kind of Mg site, one kind of Al site, and four kinds of H sites considering different site occupancies in crystallology. Doping can be an effective strategy to improve the structural and properties of pure material. In this work, seven kinds of common elements (Li, B, C, Na, Si, K, Ca) that are close to Mg or Al in the periodic table have been designed as dopants considering both the Mg site doping and the Al site doping. All fourteen kinds of cases have been taken into consideration, and the results are shown in Table 1. The thermodynamic tendency or trend of the doping can be determined by formation energy, which can be calculated by the following formula [29]:

*E*_form_ = *E*_T_(D) + *μ*_h_ − *E*_T_(P) − *μ*_d_ (1)

In this formula, the *E*_T_(D) and *E*_T_(P) stand for the total energies of the doped and pure structures while the *μ*_h_ and *μ*_d_ mean the atomic potential of the host and dopant atoms respectively.

As is shown in Table 1, it is interesting to find that the considered metallic doping elements tend to occupy the Mg site, while other non-metallic elements tend to occupy the Al site. The lattice parameters of the doped materials by various elements can be seen in Table S1 of Supplementary Materials. These thermodynamic determinations lay important foundations for all the following electrochemical calculations. As is known, the conversion-type Li-ion battery electrode works based on the electrochemical conversion reaction. For the electrode material, the specific capacity, average voltage, volume change, and electronic behavior are some basic properties to weigh whether the material can be a good candidate or not. The calculations of properties of both intrinsic and various doped Mg(AlH_4_)_2_ solids are based on the following conversion reactions (the X stands for the doping element):

Mg_8_(AlH_4_)_16_ + 64Li^+^ + 64e^−^ = 64LiH + 8Mg + 16Al (2)

Mg_7_X(AlH_4_)_16_ + 64Li^+^ + 64e^−^ = 64LiH + 7Mg + X + 16Al (3)

Mg_8_Al_15_XH_64_ + 64Li^+^ + 64e^−^ = 64LiH + 8Mg + X + 15Al (4)

**Figure 2:**
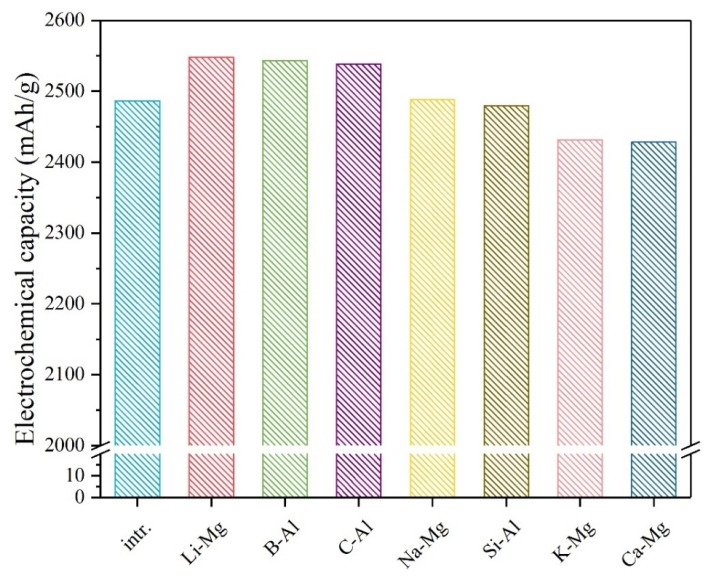
The calculated electrochemical specific capacities of the intrinsic and various doped Mg(AlH_4_)_2_ materials, e.g., the “Li–Mg” symbol stands for the Li-doped system in which the Li dopant prefers to occupy the Mg site of the Mg(AlH_4_)_2_ solid.

Figure 2 shows the electrochemical specific capacity comparison of the intrinsic and various doped systems. The theoretical capacity can be calculated by Faraday’s law:

The specific capacity = *nF*/3600*M* (5)

In this formula, *F* means the Faraday constant; *n* means the number of electrons in the above conversion reactions and *M* means the molar mass of the structure. The theoretical specific capacity of the pure Mg_8_(AlH_4_)_16_ is 2486.59 mAh/g involving 64 electrons in the electrode conversion reaction. According to Figure 2, when the Li, B, or C element dopes the system, the electrochemical capacity rises obviously, in which the Li-doped material has the highest capacity (2547.64 mAh/g). The Ca-doping is found to decrease the capacity of the pure material most.

The average voltage is another important property to be considered especially for conversion anode applications. In this work, the average voltage (versus Li^+^/Li^0^) of the conversion electrode material is calculated based on the earlier method [30,31,32]:

The average voltage V ≈ −ΔG/64F(6)

**Figure 3:**
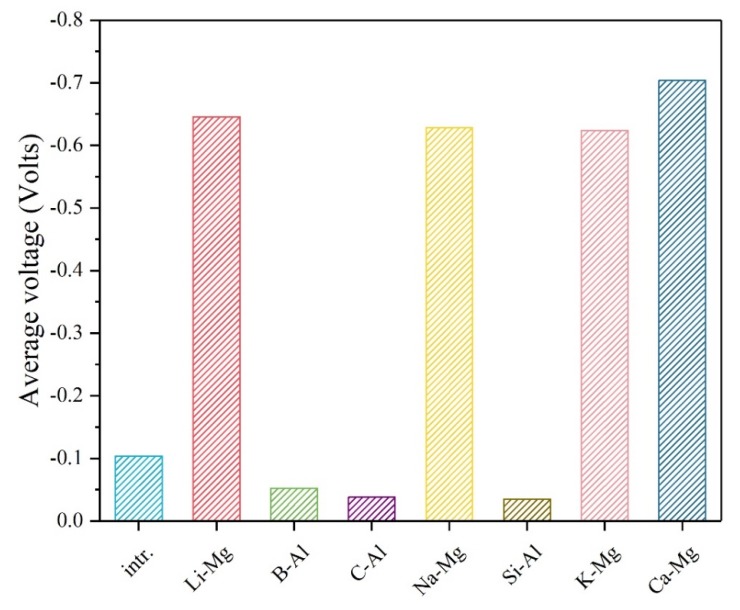
The average voltages (versus Li^+^/Li^0^) of the intrinsic and various doped Mg(AlH_4_)_2_ materials.

In this formula, *F* is the Faraday constant and −Δ*G* stands for the Gibbs free energy change after the conversion reactions shown in above (2), (3) and (4). Figure 3 gives the calculated average voltage of the intrinsic and various doped Mg(AlH_4_)_2_ materials. It is found that the investigated metal dopants (Li, Na, K, and Ca) in this work can decrease the voltage obviously. The Ca-doped structure has the lowest average voltage. Besides the electrochemical specific capacity and the average voltage, the volume change of the conversion electrode material has also been calculated. As is known, if the volume of the electrode changes too much before and after conversion reactions, it may lead to awful results for the working battery. The following formula has been used in calculations:

The volume change = (*V*_p_ − *V*_r_) / *V*_r_ × 100% (7)

**Figure 4:**
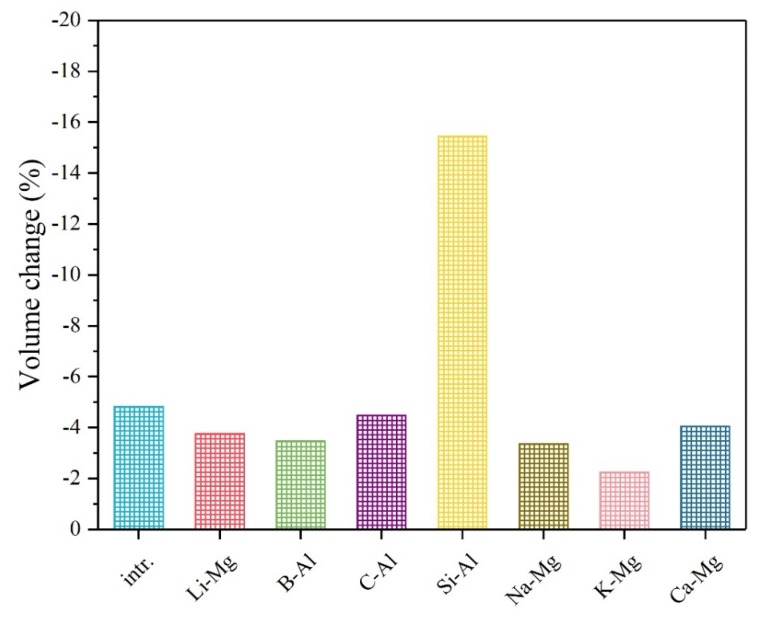
The volume change comparison of the intrinsic and various doped systems.

In this formula, the *V*_r_ stands for the volume of reactants and the *V*_p_ stands for the volume of reaction products of the above conversion reactions respectively. Figure 4 shows that most of the doping elements in this work can reduce the volume change of the intrinsic system, while the Si dopant is detrimental to the volume change of the pure material. In contrast, the K dopant can reduce the volume change most from the pristine −4.82% to −2.24%.

**Figure 5:**
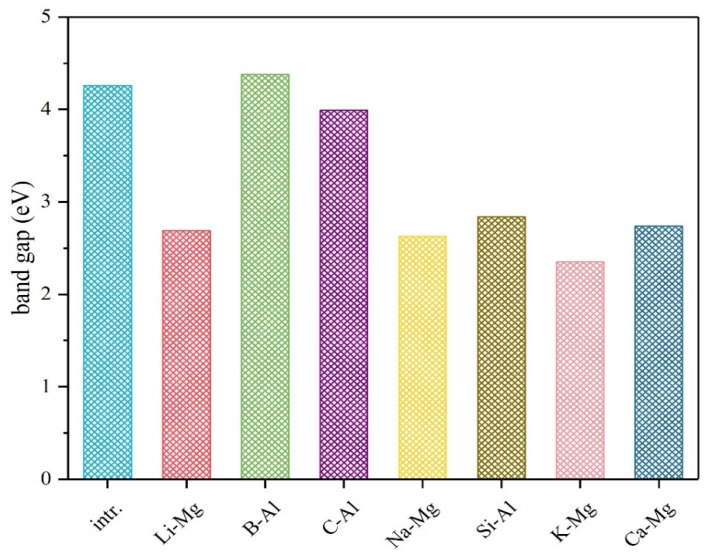
The electronic band gaps of the intrinsic and various doped Mg(AlH_4_)_2_ materials.

Figure 5 shows the calculated electronic band gaps of various systems, which are also important properties that closely relate to the electronic conductivities of the electrode materials. In this work, it is found that nearly all the dopant elements, except the B element, can reduce the bandgap of the pure material. Among these elements, the Li dopant (from pristine 4.258 to 2.687 eV), the Na dopant (from 4.258 to 2.672 eV) and the K dopant (from 4.258 to 2.348 eV) perform obviously better than the other elements.

**Figure 6:**
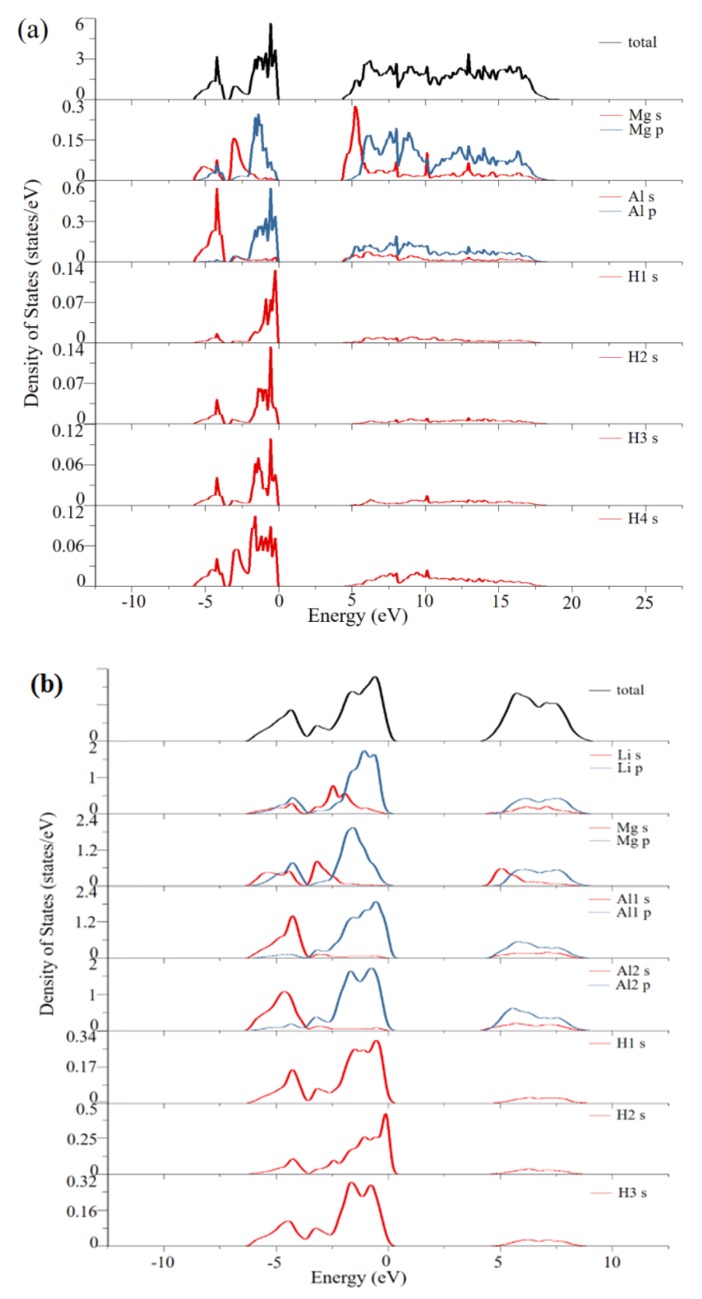
The total and partial electronic density of states (DOS) of (**a**) pure and (**b**) Li-doped Mg_8_(AlH_4_)_16_ materials. The Fermi level is set at zero; s-state is in red; p-state is in blue and the total DOS is in black.

**Figure 7:**
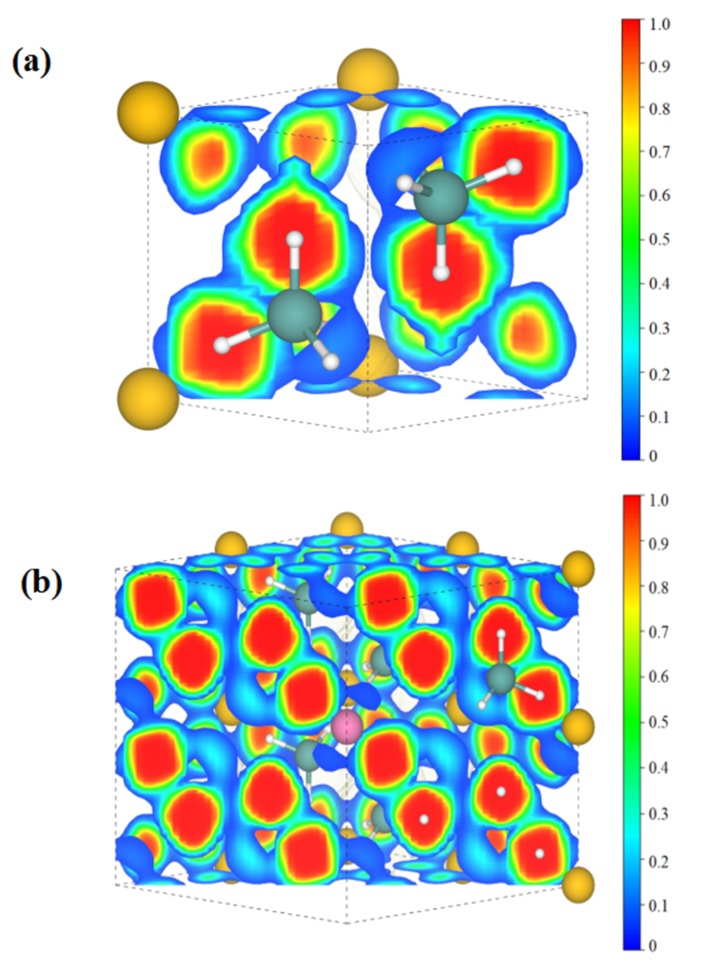
The calculated electron localization function (ELF) of the (1 1 0) plane for (**a**) pure and (**b**) Li-doped Mg_8_(AlH_4_)_16_. The Mg atoms are in yellow, the Al atoms are in green, the H atoms are in white, and the Li atom is in pink.

The above results provide comparisons of various doped systems in terms of specific capacity, average voltage, volume change, and electronic bandgap. Through this screening, it is regarded that the Li-doped system has the most comprehensive properties for conversion-type lithium storage applications compared with the other dopants in this study. In order to understand this system more deeply, we have further revealed the electronic structures through DFT calculations. Figure 6 shows the total and partial electronic density of states (DOS) of the pure and Li-doped Mg_8_(AlH_4_)_16_ materials. In Figure 6a, the total DOS can be divided into two parts. The part below 0 eV corresponds to the valence band. This part of the total density of states is mainly contributed by the Al s-state, Al p-state, and H s-state. The part above 4.258 eV corresponds to the conduction band and this part of the total density of states is mainly contributed by the Mg p-state and Al p-state. Furthermore, the hybridization of the Al p-state with H s-state leads to a strong interaction between them. The calculated band gap is close to the previous study [33]. As for Figure 6b, the Al atoms are found to have two different sites (Al1 and Al2) due to the influence of the Li dopant. In the total density of states, the valence band near the Fermi level is mainly contributed by the Al2 p-state, H s-state, and Li p-state. The conduction band near the Fermi level is mainly contributed by the Mg s-state and Al2 p-state. The Li p-state exhibits a strong interaction with the H1 s-state to form stable bonding. The H3 s-state shows a trend to hybrid with the Al2 p-state. The bandgap of the Li-doped material has decreased from the pristine 4.258 to 2.690 eV. The electron localization function of the pure and Li-doped Mg_8_(AlH_4_)_16_ structures are also calculated and shown in Figure 7. The strong covalent bonds exist between the atoms of Al and H and the ionic bonding exists between Mg and the AlH_4_ moiety. The introduced Li doping atom occupies the pristine position of Mg atom and interacts with the H atom to form some bonding. This would weaken the covalent bonding between Al and H in AlH_4_ moiety and destabilize the hydride, which can help to improve the electrode conversion reaction. Similarly, we have also calculated the density of states and the electron localization functions of both the Na-doped system and the K-doped system, which can be seen in Figures S1–S4 of Supplementary Materials. It is believed that they would also advance the in-depth understanding of metal-doped Mg(AlH_4_)_2_ systems.

## 4. Summary and Outlook

In summary, we have investigated the pure and various doped Mg(AlH_4_)_2_ systems for conversion-type lithium storage applications via first-principles design. The most thermodynamically stable doping sites in Mg(AlH_4_)_2_ for each doping element (Li, Na, K, Ca, B, C, or Si) are firstly determined. After that, the conversion-type lithium storage properties including the electrochemical specific capacity, average voltage, volume change, and the electronic bandgap are predicted based on those element-doped structures. The Li-doped system is screened to be the most acceptable candidate to improve the comprehensive properties of the pure material. In addition, the electronic structures of the pure and Li-doped Mg(AlH_4_)_2_ solids are also unveiled to deepen understanding of their properties. This ab initio study is proposed to help the design of better light-metal based hydrides for conversion electrode applications.

## Figures and Tables

**Table 1:** The calculated formation energies (eV/f.u.) of different substitutional doping elements and sites for Mg(AlH_4_)_2_.

Doping Element	Mg Site	Al Site
Li	0.002	2.439
B	−0.410	−0.757
C	−0.430	−0.870
Na	0.140	3.984
Si	−0.301	−0.895
K	0.177	4.032
Ca	−0.461	0.720

## Supplementary Materials

The following are available online at https://www.mdpi.com/1996-1944/12/16/2599/s1, Table S1: The lattice parameters of the intrinsic and doped materials by various elements, Figure S1: The total and partial electronic DOS of Na-doped Mg_8_(AlH_4_)_16_, Figure S2: The calculated electron localization function (ELF) of the (1 1 0) plane for Na-doped Mg_8_(AlH_4_)_16_. The Mg atoms are in yellow, the Al atoms are in green, the H atoms are in white and the Na atom is in dark green, Figure S3: The total and partial electronic DOS of K-doped Mg_8_(AlH_4_)_16_, Figure S4: The calculated electron localization function (ELF) of the (1 1 0) plane for K-doped Mg_8_(AlH_4_)_16_. The Mg atoms are in yellow, the Al atoms are in green, the H atoms are in white and the K atom is in purple.
