# Supplementary material for: Ab Initio Screening of Doped Mg(AlH4)2 Systems for Conversion-Type Lithium Storage
Source: Materials (Basel). 2019 Aug 15;12(16):2599. doi: 10.3390/ma12162599 (PMC6719994; doi:10.3390/ma12162599)
Supplement: Supplementary file 1 [file materials-12-02599-s001.pdf]

Supplementary Materials

Ab initio Screening of Doped $Mg(AlH_4)_2$ Systems for Conversion-type Lithium Storage

Zhao Qian [1,*], Hongni Zhang [2], Guanzhong Jiang [1], Yanwen Bai [1], Yingying Ren [1], Wenzheng Du [1] and Rajeev Ahuja [3]

[1] Key Laboratory for Liquid-Solid Structural Evolution and Processing of Materials (Ministry of Education), Shandong University & Shenzhen Institute of Shandong University, Shenzhen 518057, China
[2] Shandong Management University, Jinan 250100, China
[3] Condensed Matter Theory Group, Department of Physics and Astronomy, Ångström Laboratory, Uppsala University, SE-75237 Uppsala, Sweden
* Correspondence: qianzhao@sdu.edu.cn (Z. Qian)

Table S1. The lattice parameters of the intrinsic and doped materials by various elements.

	a(Å)	b(Å)	c(Å)	α(°)	β(°)	γ(°)	V(Å^3)
Pure	10.35	10.35	11.75	90.00	90.00	120.00	1089.81
Li	10.37	10.37	11.38	90.00	90.00	120.00	1059.73
B	10.28	10.28	11.56	90.00	90.00	120.00	1058.01
C	10.39	10.39	11.54	90.00	90.00	120.00	1079.89
Na	10.44	10.44	11.35	90.01	89.99	120.00	1071.18
Si	11.40	11.40	12.30	90.00	90.00	120.00	1383.85
K	10.54	10.54	11.23	90.01	89.99	120.00	1080.41
Ca	10.51	10.51	11.43	90.01	90.00	120.00	1092.83

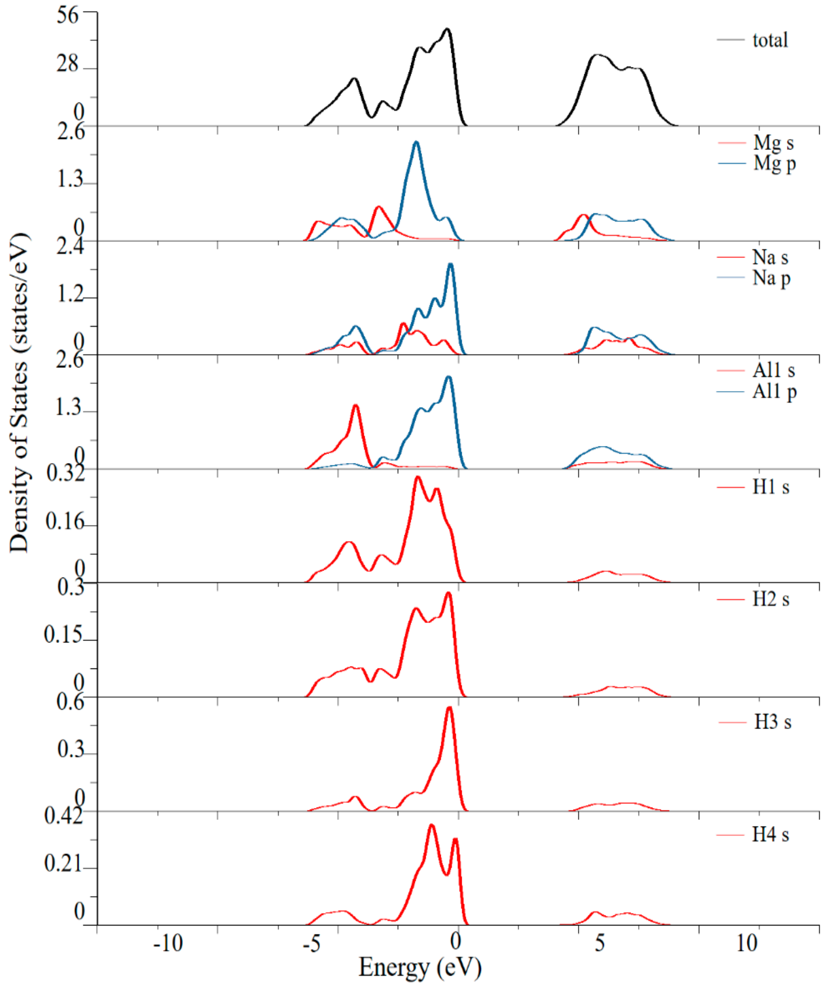

Figure S1. The total and partial electronic DOS of Na-doped $Mg_8(AlH_4)_{16}$.

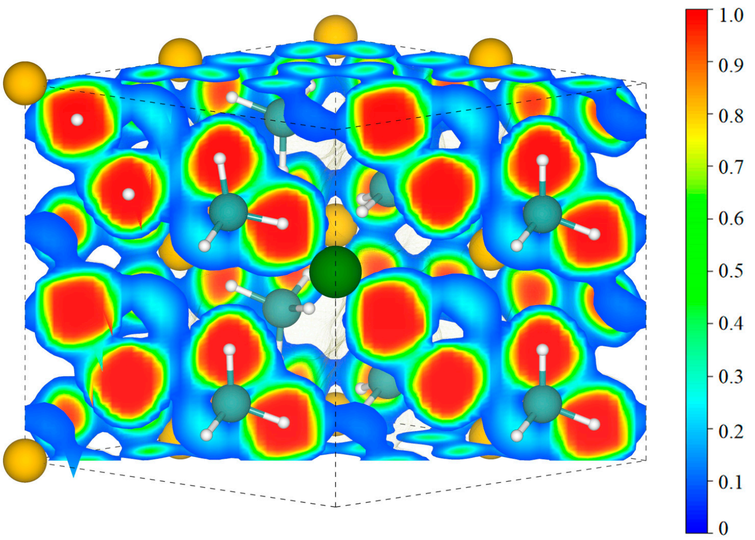

Figure S2. The calculated electron localization function (ELF) of the (1 1 0) plane for Na-doped $Mg_8(AlH_4)_{16}$. The Mg atoms are in yellow, the Al atoms are in green, the H atoms are in white and the Na atom is in dark green.

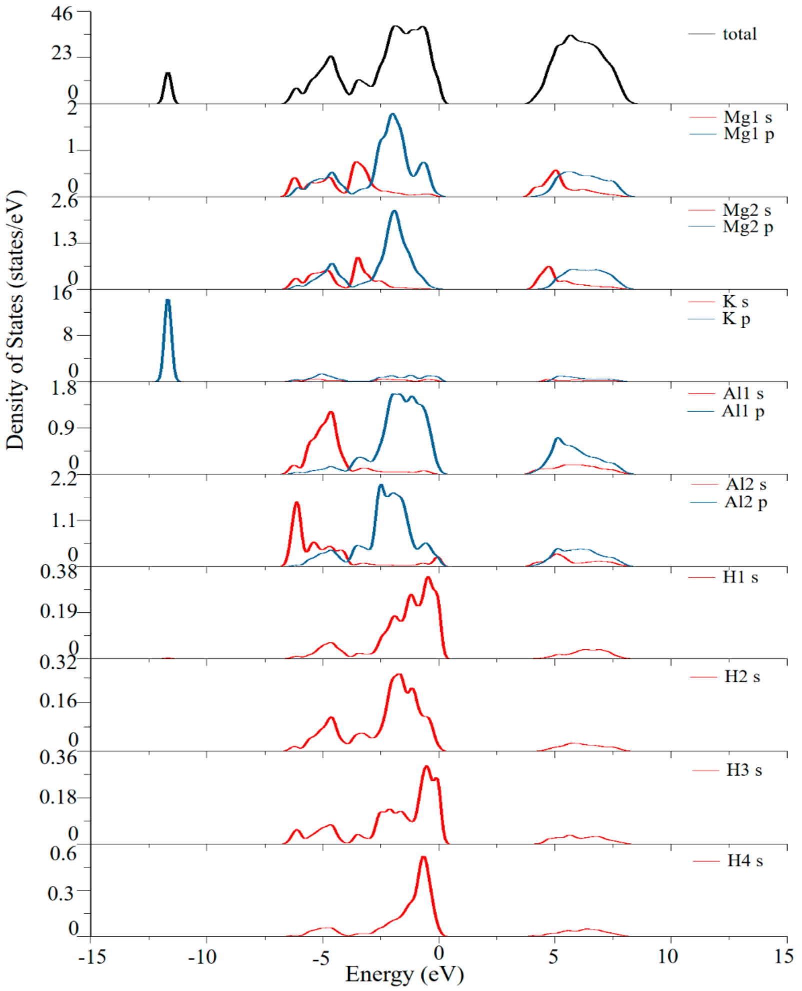

Figure S3. The total and partial electronic DOS of K-doped $Mg_8(AlH_4)_{16}$.

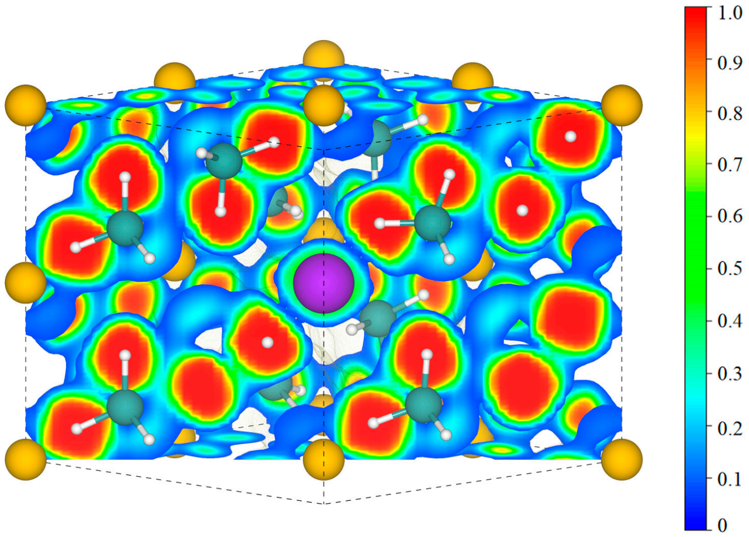

Figure S4. The calculated electron localization function (ELF) of the (1 1 0) plane for K-doped $Mg_8(AlH_4)_{16}$. The Mg atoms are in yellow, the Al atoms are in green, the H atoms are in white and the K atom is in purple.
